# Advances in Cardiac Resynchronization Therapy

**DOI:** 10.19102/icrm.2019.100604

**Published:** 2019-06-15

**Authors:** Asif Jafferani, Miguel A. Leal

**Affiliations:** ^1^Department of Medicine, University of Wisconsin School of Medicine and Public Health, Madison, WI, USA

**Keywords:** Adaptive pacing, cardiac resynchronization therapy, leadless CRT systems, multipoint pacing

## Abstract

The development of cardiac resynchronization therapy (CRT) has been crucial in reducing morbidity and mortality in patients with advanced heart failure. However, a significant proportion of patients who receive CRT fail to derive significant clinical benefits from this therapy. Successful CRT depends on a multitude of factors, including appropriate patient selection, left ventricular lead positioning, and postimplant management. Newer device-based algorithms, multipoint ventricular pacing, and the development of leadless CRT devices constitute important facets of both the present and near-future evolution of this therapy.

## Introduction

Cardiac resynchronization therapy (CRT) has acquired a pivotal role in the treatment of heart failure (HF), with associated reductions demonstrated for both mortality and morbidity in eligible patients.^1^ Its practice and concept have undergone significant developments since its introduction a quarter of a century ago.^2^ This review will focus on current concepts regarding CRT delivery and the exciting future developments that will hopefully allow us to better care for patients with HF.

## Physiological basis of resynchronization

It has been long-recognized that some patients with advanced HF also develop diseases of the intracardiac conduction system, which in turn prevent the rapid transmission of electrical impulses to all parts of the ventricles. Thus, some segments of the left ventricle (LV), for instance, may contract in a dyssynchronous fashion when compared with other regions, with examples of such being the known delay in the depolarization and subsequent contraction of the LV free (lateral) wall seen in patients with left bundle branch block (LBBB). This phenomenon decreases the overall efficiency of global LV contraction.

CRT aims to improve (or, in some cases, restore) the synchrony of the ventricular contraction, thereby improving pump efficiency. Over time, CRT aims to increase LV contractility, stroke volume, and ejection fraction and, in some cases, can induce mechanical reverse remodeling.^3^ While precise cellular and biochemical mechanisms remain incompletely understood, CRT has been shown to reverse many of the changes that have been noted during periods of dyssynchrony as well as reduce the levels of both clinical and experimental HF biomarkers.^3^

Optimal CRT delivery has been demonstrated to reduce HF hospitalizations and patient mortality and improve patient quality of life.^4–8^ However, about one-third of eligible patients do not receive the intended benefits of CRT.^9^ Therefore, active basic, translational, and clinical research is currently being undertaken in an effort to determine how appropriate patient selection, LV lead placement optimization, and postimplantation device programming and patient care can potentially maximize CRT benefits. Ultimately, restoring conduction to near-normal physiology is the primary aim and, in a select group of patients with suitable anatomy free of infra-Hisian disease, His-bundle pacing could also be applied to achieve this aim.^10,11^ This, however, is an interesting separate topic and will not be a part of the present review.

## Trial data and guidelines review

**Figure 1:**
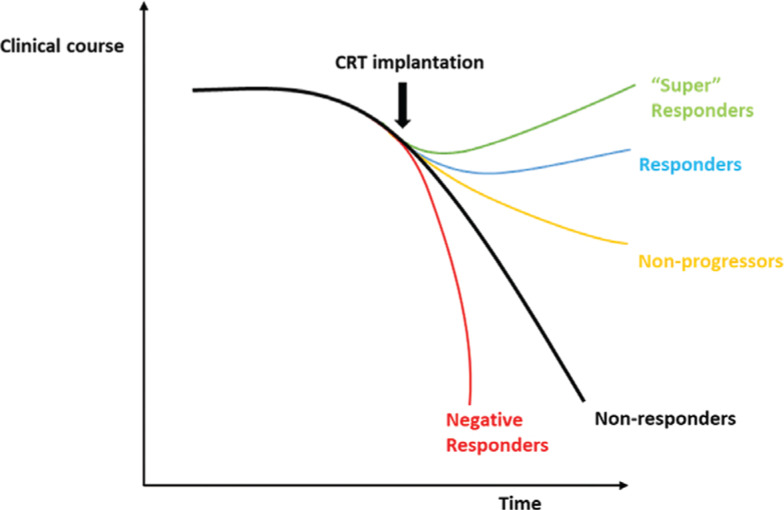
CRT response depends upon several variables, and the clinical course of patients can significantly vary. Responders experience an improvement in hard outcomes as well as quality of life measures, whereas some patients remain nonresponders and follow the expected clinical course of their primary cardiomyopathy. Response exists on a continuous scale, with super-responders experiencing a near-normalization of LV function; nonprogressors, who do not follow the expected clinical trajectory of their primary cardiomyopathy, not drawing out the complete CRT benefit; and negative responders experiencing a worsening in their clinical course following CRT implantation, respectively.

CRT response is predicated on modifying the natural history of HF and may actually represent a spectrum of response levels from near-normalization of the LV contractile function, termed a “super-response,” to worsened survival and LV function post–CRT implant, termed a “negative response”^12^ **(Figure 1)**. Historically, an array of endpoints were originally used to show CRT response including surrogate echocardiographic and physiological variables that include LV ejection fraction (LVEF), LV end-systolic volume (LVESV), LV end-diastolic diameter (LVEDD), and peak oxygen consumption (VO_2_)^13–16^; subsequently, from 2004 to 2005, larger clinical trials indicated benefits attributable to CRT in the reduction in mortality and/or HF-related hospitalizations **(Table 1)**. In all, the evidence gathered from these trials informed the current American College of Cardiology (ACC) clinical practice guidelines,^17^ as summarized in **Table 2**.

## Patient selection

Criteria for appropriate patient selection can be derived from the currently accumulated evidence. Overwhelmingly, certain electrocardiogram (ECG) criteria that indicate ventricular dyssynchrony and the clinical assessment of HF severity consistently show the best predictive ability for the prediction of response to CRT. These and other novel criteria for predicting CRT response continue to be studied and are summarized henceforth.

### Electrocardiogram criteria

The QRS complex width and a morphology pattern suggestive of LBBB appear to be the most powerful predictors for CRT response. While trials have shown a benefit of CRT in patients with a QRS complex duration of greater than 120 ms to 150 ms, a meta-analysis by Sipahi et al.^18^ showed that CRT in patients with a QRS duration of more than 150 ms was associated with a reduction in composite clinical endpoints such as death or hospitalization, while CRT in patients with a moderately prolonged QRS complex duration (120–149 ms) did not confer the same benefit. Similarly, in another meta-analysis, the presence of a LBBB pattern on surface ECG was the only strong predictor for a reduction in clinical endpoints as compared with the presence of non-LBBB conduction abnormalities.^19^ Therefore, while patients with moderately prolonged QRS or non-LBBB morphology may be considered for CRT, the strength of evidence suggesting a good response remains weaker^17^ and so other criteria should be used to predict CRT response.

It has been hypothesized that a sufficiently wide QRS complex would reflect a delay even in the left bundle in a patient with a right bundle branch block pattern, which would make CRT effective.^20^ The presence of a bifascicular block was, however, not predictive of CRT benefit in the Multicenter Automatic Defibrillator Implantation with Cardiac Resynchronization Therapy (MADIT-CRT) trial.^21^ CRT is not indicated in patients with a QRS complex duration of less than 120 ms and may even cause harm in such scenarios.^17,22^

Other ECG markers, such as P–R-interval prolongation (PRp), have also been studied as potential indicators of CRT response.^23^ In a recent retrospective analysis of 197 consecutive patients with a PRp of less than 200 ms prior to CRT implantation, PRp was independently associated with worsened outcomes of death or the need for advanced HF therapies.^24^ PRp was strongly associated with adverse outcomes, particularly in LBBB-morphology patients, and remained an independent predictor of adverse outcomes regardless of the QRS complex duration in patients with LBBB morphology.

### Heart failure severity

Most of the evidence for the benefits of CRT comes from trials that enrolled New York Heart Association (NYHA) functional classes III and IV patients.^4,13^ While subsequent trials showed a response to CRT in less-sick patient cohorts, such as NYHA functional classes I and II patients,^7,8^ the evidence remains strongest for patients with symptomatic HF (NYHA classes II–IV). There are, however, some specific patient scenarios in which earlier implementation of CRT may be considered. Needless to say, any evidence of an added benefit from CRT in patients with a clinical diagnosis of advanced HF has to start from the principle that optimal medical therapy has previously been adopted and maintained over time.

### The role of cardiac imaging

Hypothetically, the electrical dyssynchrony seen with the prolongation of QRS complex duration and morphology changes underpins mechanical dyssynchrony, leading to ineffective LV function.^20^ CRT aims to rectify this issue by restoring intraventricular and interventricular synchrony.^25^ Thus, it was a natural side effect of this understanding to also evaluate the utility of CRT in patients with mechanical dyssynchrony in the absence of electrical dyssynchrony—in other words, in those with a normal QRS complex duration and/or morphology. Three large trials, however, did not show any benefit with the use of CRT in patients with mechanical dyssynchrony upon assessment with cardiac imaging.^22,26,27^ Furthermore, one of these trials actually showed a potential for harm with CRT use in patients with a QRS complex duration of less than 130 ms and echocardiographic evidence of mechanical dyssynchrony.^22^

The utility of imaging markers, such as echocardiographic parameters, to assess dyssynchrony was initially called into question after the publication of the Predictors of Response to CRT (PROSPECT) large international observational study.^26^ This trial aimed to assess 12 predefined echocardiographic parameters regarding their ability to predict clinical and echocardiographic responses to CRT. Accordingly, 498 patients in 53 clinical centers from around the world who were eligible to undergo CRT implantation according to published guidelines had preprocedure echocardiogram recordings taken to assess, in a blinded manner, conventional and tissue Doppler imaging–based parameters. Thereafter, patients were followed for six months to discern whether any improvement occurred in a clinical composite score as well as whether there was any reduction in their LVESV. Ultimately, the ability of these parameters to predict benefit with CRT remained modest at best, with significant interobserver variability noted in the analysis of dyssynchrony parameters despite specific training.

Following the PROSPECT trial, while interest in utilizing imaging-based parameters to assess dyssynchrony with the goal of predicting CRT response has waned somewhat, smaller studies continue to show some degree of utility of imaging parameters in predicting CRT response. A recent study by Wang et al. revealed that, in a cohort of 80 patients, LBBB contraction pattern identified via radial or longitudinal strain methods using speckle-tracking predicted reverse remodeling at six months.^28^ It also increased the predictive value of a statistical risk model already incorporating QRS duration and ischemic etiology to predict reverse remodeling. Similarly, Fournet et al. in their pilot study discussed the potential role of analyzing three-dimensional strain curves using automated quantification by way of computerized algorithms to predict CRT response.^29^ Tao et al. separately shared their observations upon using gated single-photon-emission computed tomography myocardial perfusion imaging to assess both scar burden and contractility pattern, with a U-shaped contractility pattern significantly associated with LVEF improvement versus a non-U-shaped contractility pattern.^30^ Similarly, cardiac magnetic resonance imaging has also been effective in assessing scar burden, which is associated with a reduced response to CRT, as well as global ventricular dyssynchrony pattern.^31^

Finally, of interest is the fact that dobutamine stress echocardiography (DSE) can be applied to assess myocardial functional reserve. Hence, its utility in appropriate patient selection for CRT has also been studied to some extent. In a meta-analysis of nine observational studies with a total of 767 patients, Kloosterman et al. concluded that contractile reserve assessment via DSE had a significant association with CRT response.^32^ Though the analysis had evidence of publication bias, an imputation of missing data (virtual studies) still maintained the predictive association between contractile reserve and CRT response with an odds ratio of 2.42 (95% confidence interval: 1.17–5.05). Furthermore, there is additional evidence from Vukajlovic et al. that DSE can also help identify potential individuals likely to experience near-normalization of LV function, i.e., “super-responders.”^33^ However, this enthusiasm is tempered by the unavailability of evidence of an improvement in hard outcomes (e.g., major adverse cardiac event rates) in borderline candidates who do not meet conventional CRT criteria as defined by established guidelines and therefore must be balanced with implantation and long-term risks of the deployment of resynchronization device therapy.

### Other factors and the role of risk score calculators for the prediction of CRT outcomes

Among other patient-related characteristics, women have almost consistently shown a better response to CRT than men.^34^ In a MADIT-CRT trial substudy conducted among patients who were deemed to be super-responders (defined in the study as those in the top quartile for LVEF change), female sex was the second strongest independent predictor of super-response (odds ratio: 1.96).^35^ The other predictors in the order of strength of association were the presence of a LBBB pattern on surface ECG, no prior history of myocardial infarction, a QRS complex duration of 150 ms or more, a body mass index of less than 30 kg/m^2^, and a smaller baseline left atrial volume index.

Similarly, in a meta-analysis by Yin et al. that analyzed 11 observational studies including 149,259 patients, women experienced lower all-cause mortality and better improvement in echocardiographic parameters than did men.^36^

An analysis of the role of CRT in patients with atrial fibrillation (AF) and for patients requiring antibradycardia pacing is presented separately at the end of this review.

Finally, due to the abundance of risk factors and other predictors summarized above, risk scores have been created in an effort to better predict the individual patient’s clinical response to CRT. While evidence of their usefulness remains limited beyond that from the initial reporting centers, studies have continued to show some indication of their ability to predict CRT response.

In 2014, Brunet-Bernard et al. reported the development of a seven-point scoring system called L2ANDS^37^ with the following items: LBBB (two points), age older than 70 years (one point), cardiomyopathy of nonischemic etiology (one point), an LVEDD of less than 40 mm/m^2^ (one point), and the presence of septal flash (two points). Their study reported a predictive accuracy of 0.75 (C-statistic) via a cohort of 45 patients demonstrating a greater-than-15% improvement in LVESV as assessed at six months post–CRT implant. A more recent follow-up study authored by the same group included 275 patients followed for two years and showed a predictive accuracy of 0.78 (C-statistic) for CRT response, which was defined as an improvement in LVESV and freedom from major cardiovascular events (eg, death, transplantation, need for a ventricular assist device).^38^ This scoring system, however, has not yet been validated in other populations beyond the initial study sites and so its utility in clinical practice remains undefined.

## Device implantation

### Left ventricular lead placement

Optimal LV lead placement is another factor that can significantly impact the amount of benefit derived from CRT. Coronary sinus (CS) anatomy can be highly variable, with procedural difficulty frequently encountered in cannulating the CS and in landing the lead in a stable position to promote appropriate capture of the LV. This usually is possible through cannulation of a highly variable posterolateral, lateral, or anterolateral branch of the CS between the middle cardiac vein and the anterior interventricular vein.^39^ However, this can be particularly challenging in a right atrium that is either very small or enlarged with significant tricuspid regurgitation or in patients with a persistent left-sided vena cava. Particularly in these cases, preprocedure planning via imaging with computed tomography or magnetic resonance technology or intraprocedure planning using fluoroscopy may help to better define optimal positioning of the LV pacing lead.^39^ Guidelines for step-by-step procedures recommended for LV lead implantation are beyond the scope of this paper; however, it is important to note that rates of successful LV lead implantation in major previous CRT trials were around 90%,^7,8^ with challenging CS anatomy being the most common reason for implantation failure. Furthermore, LV lead dislodgement rates have been found to be around 6% in clinical trials,^40^ which again is the most important procedural complication. A wide variety of specialized tools have been developed to facilitate LV lead implantation, and it is important for the operator to be at least familiar with less frequently applied techniques and equipment like venoplasty or active fixation leads, as these can be useful in select cases such as venous occlusion or dissection. If all else fails, surgical LV lead implantation may be considered via thoracotomy, video-assisted thoracic surgery, or robotic approaches. These methods do have their limitations in achieving a suitable posterolateral lead position and in some cases have been reported to lead to higher LV lead failure rates.^39^

Broadly, three methods have emerged for targeted LV lead placement to maximize CRT benefit: anatomic distance, electrical delay, and/or mechanical delay. Additionally, the use of multipoint pacing LV leads continues to promote an evolution in our understanding of optimal LV lead placement and pacing vectors.

Intuitively, maximizing the distance between the LV and the RV leads would potentially lead to the largest region of ventricular capture for the optimal delivery of CRT, an observation that also agrees with findings in previous clinical studies.^41^ Furthermore, a MADIT-CRT trial substudy showed that apical positioning of the LV lead was less favorable,^42^ in part due to the nonphysiological activation sequence of the LV and also by further reducing the area of the ventricular myocardium activated via CRT. However, beyond these “rules,” larger studies have failed to show any significant differences in response between the anterior, posterior, and lateral lead positions,^39^ with a slight advantage reported when the LV pacing lead was positioned in a posterolateral or lateral CS tributary.

A variety of algorithms have been developed to identify the site of the latest electrical activation using the timing of the local LV depolarization wave recorded during lead implantation. The difference between the start of the QRS complex as measured via surface ECG to the local electrogram (also called the Q–LV interval) has been used in many of these algorithms to determine the optimal site for implantation of the LV lead. In general, the longer this delay period, which is indexed to the QRS width (also called the LV electrical delay or CS delay index), then the better the responses to CRT are, according to smaller studies.^39,43^ A recent study, the ENHANCE CRT trial, failed to show the benefit of this approach to optimize CRT delivery in a small population of patients with non-LBBB QRS complex morphology.^44^

Imaging modalities can also be used to target the region of maximum mechanical delay and avoid regions with scar as part of an effort to maximize the response to CRT. In this regard, the randomized Targeted LV Lead Placement to Guide CRT (TARGET) trial assessed the utility of echocardiographic radial-strain imaging to target the site of latest mechanical activation with the avoidance of scar as compared with standard CRT placement.^45^ This trial found higher rates of clinical response in the intervention group (83% versus 65%) and lower rates of combined clinical endpoints. Similarly, the Speckle Tracking–assisted Resynchronization Therapy for Electrode Region (STARTER) trial assessed the utility of echocardiography-guided lead placement and found better event-free survival (hazard ratio: 0.48) in the intervention group.^46^

The role of magnetic resonance–based LV lead placement to avoid LV scar^47^ as well as three-dimensional echocardiography also continue to be investigated in this regard.^48^

### Multipoint (or multisite) pacing

A natural evaluation of the concept of dyssynchrony and resynchronization led to the hypothesis of using pacing from multiple sites as another alternative to better deliver CRT. Hence, multilead and/or multisite pacing strategies were evaluated and, although deemed safe in the short-term, have encountered clinically relevant problems in pragmatic terms, including a difficulty to consistently ensure multisite capture with the use of Y-adaptors and issues related to accelerated battery depletion.^20^ These factors subsequently led to the development of single-lead multipoint pacing (MMP) systems.

Generally, MPP leads have been sought after for some time, as they allow for the involvement of multiple programmable vectors, thereby decreasing the chances of undesirable outcomes such as very elevated capture thresholds or phrenic nerve stimulation. This strategy has also been shown to be associated with a reduction in mortality in a large nationwide database.^49^ More recently, the ability to use simultaneous MPP cathodes has been studied and demonstrated favorable changes with respect to hemodynamic and echocardiographic parameters following CRT implantation.^50,51^ Multiple larger trials are currently ongoing to better evaluate the safety and efficacy of MPP systems to enhance CRT response in all-comers,^52^ CRT nonresponders,^53^ and patients with narrow QRS complexes.^54^

### Postimplantation management

Postimplant device management remains critical and an optimal response to CRT depends on appropriate postprocedure programming and ongoing device optimization. A high percentage of biventricular pacing is essential for the delivery of optimal CRT: Hayes et al. reported that effective CRT present for more than 98.4% of the time is associated with better clinical outcomes.^55^ In routine clinical practice, target CRT percentages of greater than 95% are typically used.

**Figure 2:**
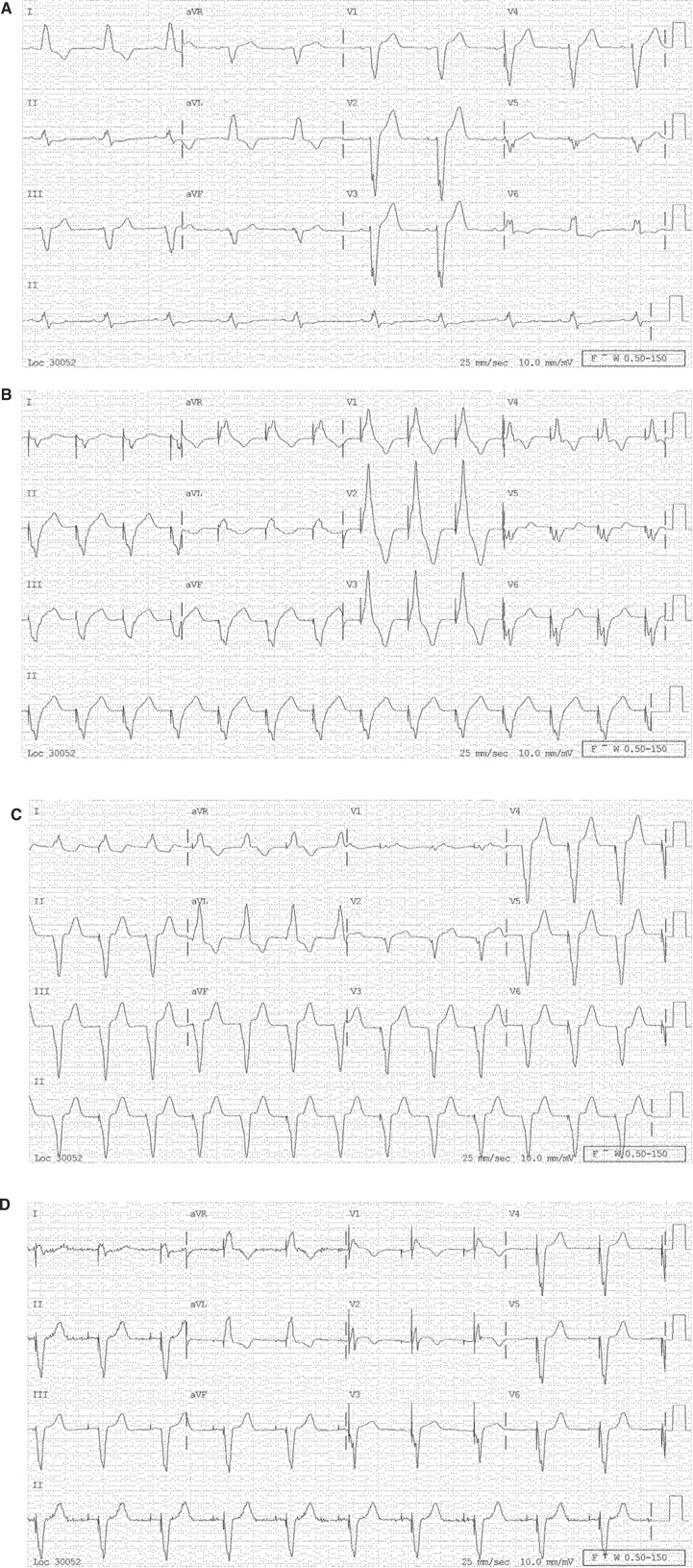
A series of 12-lead ECGs showing alterations in QRS with different activation sequences in the same patient implanted with a CRT device with changes in active pacing leads. **A:** Native QRS with LBBB morphology. **B:** Only LV pacing with RV lead deactivated. **C:** Only RV pacing with LV lead off. **D:** CRT with both RV and LV leads activated.

A 12-lead ECG is often the most useful method to detect poor (or absent) LV capture due to lead failure or dislodgement or other issues related to programming (eg, anodal capture, fusion). In general, optimal LV capture is determined by a dominant R-wave in lead V1 and a QS complex in leads I and aVL **(Figure 2)**. The absence of these features may indicate a loss of LV capture, lead malfunction or dislodgement, fusion between paced and intrinsic complexes, or LV activation delay due to possible scar or anodal capture.^20^ The LV lead capture threshold test should be performed with the RV lead turned off and with real-time ECG data acquisition occurring whenever feasible (ie, in nondependent patients) in order to better evaluate the occurrence of LV capture **(Figure 2B)**.

### The role of atrioventricular and interventricular interval optimization

**Figure 3:**
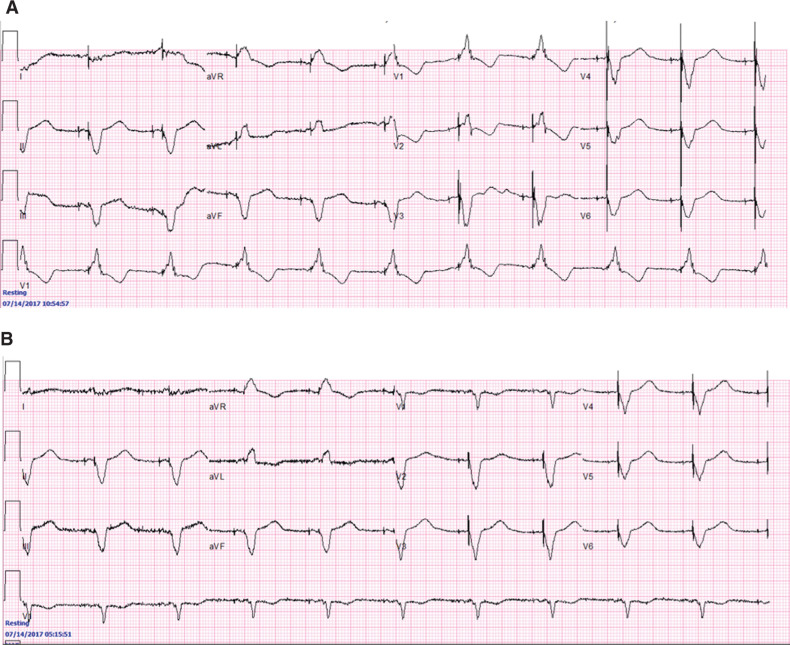
A series of 12-lead ECGs obtained in a patient at one day post–CRT implantation. **A:** Patient likely has significant scar with inadequate LV capture of initial ECG. **B:** LV lead offset by 20 ms produces better QRS morphology.

Several studies have evaluated the role of A–V and V–V interval optimization using ECG-based, echocardiography-based, or intracardiac electrogram (IEGM)–based methods in order to improve the clinical response to CRT. Iterative QRS complex–based methodologies aimed to improve the morphology of the paced QRS complexes based on optimizing AV delays and V–V intervals are commonly used **(Figure 3)**; however, echocardiography-based methodologies have been the most widely studied in the literature thus far.^25^

These methods involve algorithms that aim to optimize LV diastolic filling by assessing mitral valve inflow velocity patterns, cardiac output (stroke volume) by assessing the aortic valve pulse wave Doppler velocity–time integral, or various M-mode or tissue Doppler-derived parameters, respectively. While smaller studies have shown their usefulness in improving acute hemodynamic measures with CRT,^25^ it is unclear whether these benefits translate into persistent clinical improvement by optimizing CRT delivery over time.

Three IEGM-based algorithms are available today, with the common goal of correcting intrinsic electrical activation delays via the optimal setting of A–V and V–V intervals.^25^ These algorithms include QuickOpt and SyncAV (Abbott Laboratories, Chicago, IL, USA); SmartDelay (Boston Scientific, Natick, MA, USA); and Adaptive CRT (Medtronic, Minneapolis, MN, USA). They have been evaluated to date in a few randomized trials^56–58^ and compared with empiric interval programming and echocardiography-based methods. While these trials have demonstrated safety when using these novel algorithms, benefits involving clinical endpoints as compared with empiric device settings or echocardiography-based approaches are yet to be displayed.

It must be mentioned, however, that the Adaptive CRT study, in a post-hoc analysis, did confirm improved clinical outcomes in patients receiving LV-only pacing [ie, patients with LBBB and no atrioventricular (AV) block] versus the echocardiography-optimized arm.^58,59^ The Adaptive CRT algorithm is a continuous ambulatory-based algorithm that aims to modify the CRT delivery mode and programmed intervals based on dynamic rhythm characteristics, such as heart rate, PR interval, and the development of AV block. Due to these observations, which may support the delivery of LV-only pacing in select patients, the larger AdaptResponse trial is currently ongoing and has the goal of testing the superiority of this algorithm to reduce deaths or HF decompensation as compared with conventional CRT delivery.^60^

### Improving the cardiac resynchronization therapy percentage

Suboptimal biventricular pacing percentages are often related to conducted atrial tachyarrhythmias (ATs), frequent ventricular ectopy, or inappropriately programmed AV delays.^20^ CRT devices have the ability to trigger LV pacing in response to sensed events detected by RV leads, leading to fusion or pseudofusion complexes. These are thought to be less effective hemodynamically when compared with LV lead–initiated events. Interestingly, most CRT devices tend to overestimate the CRT percentage in this setting and do not indicate the percentage of “effective” biventricular-paced QRS complexes^61^; therefore, other ancillary methods such as ambulatory ECG recording (eg, Holter monitors) have been used to better estimate the actual CRT percentage. Here, a mention should be made about eCRTAF, which is a proprietary algorithm from Medtronic (Minneapolis, MN, USA) that can distinguish effective biventricular-paced QRS complexes from fusion and pseudofusion complexes and adjusts the pacing rate to maximize effective CRT delivery^62^; at this time, further larger studies, however, are needed to evaluate its efficacy in improving HF outcomes related to effective CRT delivery.

Premature atrial and ventricular beats also reduce the efficacy of CRT delivery. In a MADIT-CRT substudy, there was a threefold increase in the probability of low CRT pacing percentage in patients with 0.1% to 1.5% ectopic beats, which translated into an increased risk of death, HF hospitalization, and ventricular arrhythmias in patients as compared with in controls.^63^ Treatment with β-blocker agents, dedicated antiarrhythmic therapy (often class III drugs), and invasive management in selected cases (eg, catheter ablation procedures) may improve outcomes in these patients.^64^

## Special situations

### Atrial fibrillation

The role of CRT in AF and other ATs remains incompletely studied to date.^65^ At this time, there have been no trials objectively performed to test the efficacy of CRT in patients with AF; consequently, CRT use in this setting has only been given a class IIa status according to current practice guidelines.^14^ AF and other ATs pose a special challenge in CRT delivery due to the irregularity of the intrinsic rhythm, the loss of atrial contraction, and frequently noted rate control problems that limit effective biventricular triggered responses.^9,65^ Furthermore, new-onset AF is relatively common in CRT recipients, likely due to its association with advanced HF.^66–68^ The prognosis of AF in this setting remains uncertain; a MADIT-CRT substudy showed no difference in the clinical benefits obtained with CRT therapy in a cohort of patients with a history of intermittent AF/ATs versus controls.^66^ However, a post-hoc analysis of the Comparison of Medical Therapy, Pacing, and Defibrillation in HF (COMPANION) trial suggested that AF patients did not draw any greater benefit from CRT in comparison with controls.^69^

Practical recommendations for the management of such patients continue to emphasize the role of effective CRT delivery in these individuals. Guidelines and expert recommendations emphasize the role of antiarrhythmic drugs, such as amiodarone, in an effort to restore and maintain sinus rhythm, with escalation to AV nodal ablation warranted if adequate CRT pacing percentages are not achieved.^9,65^ AV nodal ablation has been shown to be effective in restoring effective CRT delivery and thereby achieving favorable clinical endpoints.^70,71^ Questions still remain regarding the role of AV nodal ablation in patients with intermittent or paroxysmal AF/ATs as compared with adequate pharmacological therapy. AV nodal ablation has its own disadvantages, including permanent pacemaker dependency and associated risks if complications arise following generator and/or lead replacement.

### Patients with bradycardia requiring real or predicted elevated right-ventricular pacing percentages

Higher percentages of right ventricular (RV) pacing have been associated with electrical and mechanical dyssynchrony, especially in patients with depressed LV systolic function at baseline.^72^ The Biventricular Versus RV Pacing in HF Patients with AV Block (Block-HF) trial was designed to test the efficacy of CRT in patients with NYHA functional classes I through III HF with a LVEF of 50% of less who were predicted to require higher percentages of ventricular pacing.^73^ CRT was effective in reducing the combined endpoint of death, acute HF exacerbation, or increase in LVESV. Hence, preemptive CRT-based device therapy is considered reasonable in such patients (baseline LVEF ≤ 50%, mild HF symptoms, and a relatively high anticipated ventricular pacing burden).^17^

## Newer advancements

### Leadless cardiac resynchronization therapy systems

After the development and subsequent approval of leadless RV pacing systems, interest has concomitantly increased regarding the development and application of a reliable leadless system capable of providing CRT.

Beyond the limitations of conventional lead systems, including the established risks of lead fracture, thrombogenicity, and potential nidus for bacteremia and infection,^74^ CRT delivery is often limited by anatomical challenges relating to the positioning of the LV pacing lead. In a transvenous CRT delivery system, this specifically includes obtaining access to the CS, whose anatomy may be highly variable across individual patients, including in some cases where the CS is atretic or does not include any tributaries deemed suitable for the implant of a pacing lead.^75^ Furthermore, higher LV pacing thresholds and phrenic nerve stimulation pose additional challenges for safe implantation of the LV lead.^76^ These have historically been dealt with by either surgically implanting epicardial leads or by placing endocardial LV leads, which both come with their own sets of unique risks and potential complications.^20^

**Figure 4:**
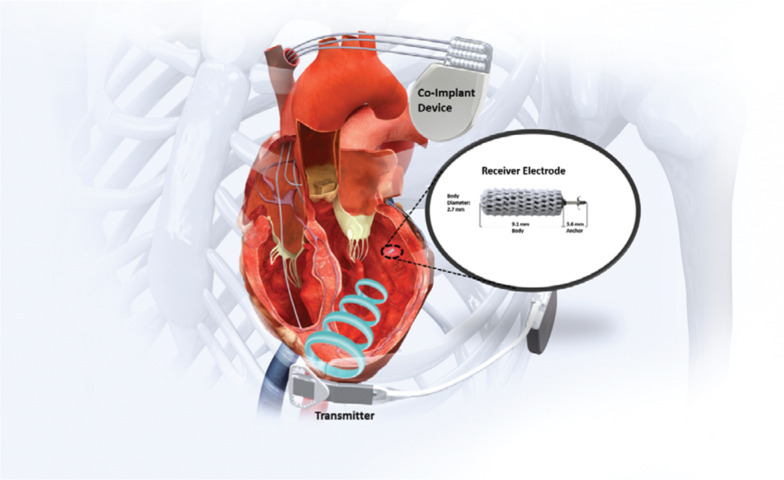
The WiSE-CRT system (EBR Systems, Sunnyvale, CA, USA) consists of a multicomponent system with a conventional right-sided pacemaker/defibrillator and an intracardiac electrode implanted in the LV. The device system has an extracardiac transmitter, which synchronizes with the RV pacing system and transmits an ultrasound-based signal to the LV electrode, which then initiates LV pacing. Figure adapted with permission from EBR Systems.

In this regard, the only leadless LV-based pacing system to undergo clinical testing to date is the WiSE-CRT system (EBR Systems, Sunnyvale, CA, USA).^77^ This is a multicomponent system that consists of a conventional right-sided dual-chamber defibrillator that communicates with a phased-array ultrasound pulse generator implanted subcutaneously in the lateral thorax. This generator transmits ultrasound energy to a small (9.1 × 2.7 mm; 0.05 cm^3^) electrode implanted in the LV endocardium **(Figure 4)**. The initial trial evaluating this system was stopped early due to a high incidence of cardiac tamponade, including one fatal event associated with the LV electrode delivery^77^; this led to modifications in the design of the delivery system with the delivery sheath now equipped with a balloon to ensure safe delivery of the LV endocardial electrode.

Subsequent experience published in the Safety and Performance of Electrodes Implanted in the LV (SELECT-LV) study included a cohort of 35 patients and showed a 97.1% success rate of LV pacing system implantation as well as a 97% rate of successful biventricular capture at one month, with 88% of the patients demonstrating improvement in a clinical composite score at six months.^78^ There were three periprocedural adverse events, including one case of ventricular fibrillation, one electrode embolization, and one vascular access–related event. There was also a 23% rate of device-related adverse events observed during the first month of follow-up. Of note, regarding the optimal anticoagulation strategy in patients who are not already candidates for systemic anticoagulation therapy due to other indications, a dual antiplatelet strategy was used in this study without any significant risk in the short follow-up period reported.

## Conclusion

CRT therapy has progressed significantly from its intuitive first concepts and applications to actually impacting in a very significant way the mortality, clinical care, and quality of life of patients with advanced HF. Currently, there remain several areas of active investigation aimed at reducing nonresponder to CRT rates and also expanding CRT’s indications to populations beyond those detailed in current practice guidelines. The impact of multipoint pacing, adaptive CRT programming optimization, and the development and use of wireless CRT systems also are areas of excitement and ongoing research, with eventual findings possibly further increasing the impact of electrical resynchronization on the care of these patients in the future.

## Figures and Tables

**Table 1:** Design and Results of Landmark CRT Clinical Trials

Trial	Patients	Follow-up Duration	Comparison Groups	NYHA Class	LVEF	QRS Duration	Primary Endpoints	Results
CARE-HF^6^	813	29.4 months	OMT versus CRT-P	III–IV	≤ 35%	≥ 120 ms	• All-cause mortality or hospitalization	• CRT-P decreased all-cause mortality and HF hospitalizations • CRT-P improved NYHA class and QoL
REVERSE^7^	610	12 months	CRT-on versus CRT-off	I–II	≤ 40%	≥ 120 ms	• Worsening of HF clinical composite response	• CRT did not improve primary endpoint but did decrease LVESVI and delayed time to first HF hospitalization
RAFT^9^	1,798	40 months	ICD versus CRT-D	II–III	≤ 30%	≥ 120 ms	• All-cause mortality or HF hospitalization	• CRT-D decreased all-cause mortality, cardiac mortality, and HF hospitalizations
MADIT-CRT^21^	1,820	12 months	ICD versus CRT-D	I–II	≤ 30%	≥ 120 ms	• All-cause mortality or HF event	• CRT-D reduced composite endpoint of all-cause mortality or HF events and improved echo parameters • CRT-D did not reduce all-cause mortality
COMPANION^70^	1,520	15 months	OMT versus CRT-P versus CRT-D	III–IV	≤ 35%	≥ 120 ms	• All-cause mortality or hospitalization	• CRT-P and CRT-D decreased all-cause mortality or hospitalizations • CRT-D decreased all-cause mortality
BLOCK-HF^74^	918	37 months	RV versus BiV pacing	I–III	≤ 50%	123–125 ms	• All-cause mortality, HF event, or ; 15% LVESV increase	• BiV pacing improved composite primary endpoint and reduced HF hospitalization

BiV: biventricular; CRT: cardiac resynchronization therapy; CRT-D: cardiac resynchronization therapy with a defibrillator; CRT-P: cardiac resynchronization therapy with a pacemaker; HF: heart failure; LVEF: left ventricular ejection fraction; LVESV: left ventricular end-systolic volume; LVESVI: left ventricular end-systolic volume index; NYHA: New York Heart Association; OMT: optimal medical therapy; QoL: quality of life.

**Table 2:** Summary of American College of Cardiology/American Heart Association Guidelines Indications for Consideration of CRT in Patients^17^

Rhythm	QRS Morphology	QRS Duration	NYHA Functional Class	Level of Recommendation
Sinus	LBBB	≥ 150 ms	II, III, ambulatory IV	Class I
		120–149 ms	II, III, ambulatory IV	Class IIa
		≥ 150 ms	I + LVEF < 30% + ischemic heart disease	Class IIb
	Non-LBBB	≥ 150 ms	III, ambulatory IV	Class IIa
		120–149 ms	III, ambulatory IV	Class IIb
		≥ 150 ms	II	Class IIb
		120–149 ms	I, II	Class III
Atrial fibrillation	Any	≥ 120 ms	III, ambulatory IV	Class IIa
Significant (> 40%) ventricular pacing		Any	I, II, III, ambulatory IV	Class IIa

LBBB: left bundle branch block; LVEF: left ventricular ejection fraction; NYHA: New York Heart Association.
